# Dietary and Hygiene-Related Knowledge Versus Reported Behaviors of Eighteen-Year-Olds: A Nationwide Cross-Sectional Study

**DOI:** 10.3390/nu17050871

**Published:** 2025-02-28

**Authors:** Dorota Olczak-Kowalczyk, Marcin Studnicki, Anna Turska-Szybka

**Affiliations:** 1 Department of Paediatric Dentistry, Medical University of Warsaw, ul. Żwirki i Wigury 61, 02-091 Warsaw, Poland; dorota.olczak-kowalczyk@wum.edu.pl; 2 Department of Biometry, Warsaw University of Life Sciences, Nowoursynowska 166, 02-787 Warsaw, Poland; marcin_studnicki@sggw.edu.pl

**Keywords:** adolescents, dietary and hygiene-related behaviors, dietary and hygiene-related knowledge, education, eighteen-year-olds, oral health

## Abstract

**Background/Objectives**: The aim of the present study was to examine eighteen-year-olds’ self-assessed knowledge about diet and hygiene, behaviors that they report, and education they receive during dental appointments. **Methods**: Questionnaires distributed among 1611 subjects included questions concerning sociodemographic factors, dietary and hygiene knowledge and behaviors, and dental visits. Spearman’s rank correlation and a bivariate and multivariate logistic regression analysis was conducted; odds ratios (ORs) and adjusted odds ratios (AORs) were calculated (*p* ≤ 0.05). **Results**: Respondents self-assessed their oral health knowledge; 63.1% of them considered it to be limited, and 40.7% understood the cariogenic effect of frequent snacking. Dentists and a dental team (DT) were the main source of information (55.7%). Dietary advice was recommended by 10.8% of dentists and the DT, while check-up appointments were advised by 49.2%. Parents’ higher education contributed to a lower intake of cariogenic food. This effect was stronger for the mother’s education. Dental appointments scheduled twice a year increased the chances of consuming healthy food (A2OR = 1.21 (1.07–2.11); *p* = 0.0028). Being informed increased the chances of toothbrushing ≥ 2 times (OR: 1.21, CI 1.10–1.46), using fluoridated toothpaste (OR: 1.26 CI1.05–1.55), and the frequency of appointments (A1OR: 1.56 CI 1.21–1.87; A2OR: 1.78 CI 1.54–1.91). **Conclusions**: Knowledge and oral health related behaviors, as well as the involvement of the dental team in education, are inadequate. Education and instruction in the dental office has a favorable influence on oral-health-related behaviors. It is, therefore, necessary to undertake systemic solutions so that dental practitioners are more involved. Providing oral health knowledge should be the standard of care.

## 1. Introduction

Positive oral-health-related behaviors, such as following a diet with low cariogenicity (avoiding foodstuffs that contain sugar), regular plaque removal (oral hygiene, brushing/flossing), the use of fluoridated toothpaste, and regular dental appointments, are oral health determinants that are important for every person, regardless of their age. In adolescence, they are particularly important not only for current oral health but also have profound consequences in the long term [1,2].

Adolescence is a period of significant psychological and behavioral changes, when teenagers develop habits that continue into adulthood. It is also the time of an increased risk associated with poor oral health, as well as greater independence, bad eating habits, low priority given to oral hygiene, and a lack of possibilities of seeking care or its avoidance [1,2,3]. The definition of adolescence has undergone a transformation due to an earlier onset of puberty, delayed beginning of role changes, and the influence of unprecedented social forces, such as social media [1]. In this demographic group, a large number of unfulfilled needs regarding oral health are recognized [4,5,6]. A recent meta-analysis has revealed that dental caries and other oral health problems remain a common burden during adolescence in the European population [7]. Due to these circumstances, there is a need to strengthen the promotion of positive oral health behaviors among teenagers. Oral health promotion and achieving better oral care in this period may bring an array of benefits thanks to the improvement of general health in the long term. However, the evidence confirming the effectiveness of education regarding oral health behaviors in adolescents or young adults is insufficient and generally understudied, with the majority of research aimed at children [2,3,8]. Many studies indicate the improvements regarding oral health behaviors that occur due to the education provided by health care workers in their daily duties [8]. It has been shown that acquisition of the knowledge regarding oral health is positively correlated with adequate dietary and oral hygiene practices, as well as an improvement in nutrition and the condition of oral health [2,9,10]. The campaigns that promoted healthy behaviors organized thus far have taken a form of indirect methods that utilized posters, pamphlets or mass media, and direct ones—group trainings at schools or individual trainings [11,12,13].

Schools and dental offices are perhaps the best places for promoting oral health because this is where young people spend most of their daytime life [14]. Schools provide an ideal setting to deliver oral health knowledge in combination with preventive services to achieve oral health promotion. A school-based approach has been reported to be more efficient in delivering preventive and curative services than a community-based approach [14]. Perhaps, school-aged adolescents are in particular need of a preventive program to ensure positive long-term dental health. The regularity of behavioral education can be ensured through dental appointments [15]. A dental team is believed to play a significant role in providing oral health knowledge, creating and promoting behaviors.

Over the last 20 years, the access to dental services in Poland has increased. Schools provide students with free dental treatment until they complete the second level of education, up to the age of 19. If there is no dental office in a particular school, such an institution is supposed to have an agreement with the nearest clinic that has a contract with the National Health Fund of Poland. This is because learners must have a place where they can get free appointments to assess their oral health and hygiene, as well as provide demonstrations and dietary advice aimed at preventing dental caries. In offices having a contract with the National Health Fund, adolescents can have a free check-up, fluoride varnish application, and professional cleaning of teeth every three months. Thus, there is an opportunity to provide oral health education. Adolescents can also benefit from the services offered by private dental surgeries. The Polish model, as part of the Eastern European model, is mainly characterized by private service, focusing on treatments instead of oral prevention and promotion strategies. Even so, epidemiological studies conducted in Poland have revealed that the incidence of dental caries and its severity remains at a high level [16,17,18]; 6.8% of eighteen-year-olds were caries-free in 2017—an increase of 4.2% compared to 2001. The mean DMFT value decreased from 7.3 to 6.5 only. Hygiene behaviors changed dramatically. In 2001, 7.1% of eighteen-year-olds brushed their teeth at least twice a day, whereas in 2017, it was 67.8% [16,18].

Even though the research on individuals at the age of 18 in Poland was conducted six times (1998, 2001, 2004, 2008, 2012, 2014), dietary habits were not the focus of the analysis of nationwide studies until 2014. In comparison with that year, the daily intake of coffee with sugar (or drinking it a few times a day) has slightly decreased (from 24.2% to 19.7%), SSBs from 27.1% to 21.5% and the consumption of sweets has remained at the same level (23.7%) [18]. We also found that another contributor to added sugar in adolescents was sweetened tea (42.6%) and sweetened juices (20%), consumed at least once or several times a day, which considerably supplies the oral environment with fermentable carbohydrates. Currently, in Poland there exists no nationwide dental education program in the field of dietary and hygiene-related behaviors geared towards eighteen-year-olds.

The aim of the study was to assess dietary and hygiene-related knowledge versus the reported behaviors of eighteen-year-olds.

## 2. Materials and Methods

The study material consisted of data obtained during a nationwide epidemiological cross-sectional study of eighteen-year-olds in Poland, conducted from October to November 2017, which were part of the Ministry of Health program “Monitoring of the oral health of the Polish population between 2016–2020” in accordance with the WHO criteria 2013. Consent of the Bioethics Committee at the Medical University of Warsaw was obtained (No KB/134/2017 of 6 June 2017).

### 2.1. Participants

The study group was randomly selected in a three-stage cluster sampling procedure: district/community, urban/rural, and high schools in all 16 provinces of Poland. High schools were chosen to ensure similar educational levels to avoid a potential risk of bias. The inclusion criteria for the study were as follows: (i) the consent of the head teacher of the educational institution; (ii) informed written consent to participate in the studies; (iii) a fully completed questionnaire. Questionnaires with missing data were excluded. Requests for signed consent from sampled adolescents, along with letters informing about the scope of the study, were distributed by the teachers. In order to maintain anonymity, a code number was assigned to each of the respondents, with which the questionnaire was marked.

### 2.2. Sample Size

The study sample size was estimated using the online sample size calculator software EPI-INFO 7.2 (Centers for Disease Control and Prevention: EpiInfo™ https://www.cdc.gov/epiinfo/index.html, accessed on 14 December 2024), with a 5% accepted margin of error and a 95% confidence level. A minimum sample size of 1200 was calculated based on the total number of eighteen-year-old adolescents living in Poland and their oral health knowledge and frequency of dental visits. Data on the total number of adolescents were retrieved from the Central Statistical Office. The overall number of eighteen-year-olds in Poland was 379,344. Based on the population of eighteen-year-olds from the special risk group, a percentage of good knowledge was 31%, and a percentage of those going to a dental office at least once a year was 47% [19]. A total of 2000 adolescents attending twenty-five high schools were originally invited to take part in the study. The number of participants exceeded the minimum sample size for a potential risk of refusal to be involved in the study. A minimum survey response rate of at least 60% was established to ensure that non-response bias did not threaten the validity of the findings. Ultimately, the study covered 1611 youths representing all 16 provinces from twenty-three high schools.

With a female-to-male ratio and urban-to-rural ratio of 1:1 in the respondents, there was minimal risk of sampling bias in the present study.

### 2.3. Questionnaire

The structured questionnaire was designed by DO-K and based on the previously used questionnaires in Polish national oral health surveys, on the relevant literature concerning oral-health-related knowledge and behaviors, on a validated food frequency questionnaire (FFQ), and on the World Health Organization (WHO) Basic Methods for Oral Health Surveys [16,17,18,19,20,21,22,23]. The questions were also adopted from the Comprehensive Measure of Oral Health Knowledge (CMOHK) questionnaire to assess the level of a respondent’s oral health knowledge [24]. The Health Belief Model (HBM) was also made use of in order to connect the relationship between self-assessed knowledge/self-awareness and behaviors and oral health outcomes as a basis for the study design [25]. The validity of the questionnaire was reviewed and tested by two pediatric dentists, and modifications to the questionnaire were implemented. The questionnaire was pre-tested and re-tested on a randomly selected sample of twenty eighteen-year-old subjects who were not included in this study. Spearman’s correlation coefficient was used to assess the reliability (r = 0.763). Modifications were made where necessary to resolve ambiguities and to avoid misinterpretation of the questions.

Questionnaire forms comprised questions on the following: (i) socio-demographic characteristics (sex, self-assessed economic status, education level of parents/caregivers of the study subjects); (ii) oral health behaviors, dietary habits (including sweets and snacks category of FFQ and the frequency of consuming sweet snacks), hygiene habits (toothbrushing frequency, intentional use of fluoridated toothpaste, oral hygiene products), fluoride prophylaxis, smoking); (iii) dental visit (the reason for the dental visit, whether they have seen a dentist in the past year, the timing of the last dental visit, frequency of dental visits and dental check-ups, payment for dental appointments); (iv) self-assessed oral health status; (v) previously received education concerning oral health as performed by the dental team; (vi) level of self-assessed knowledge about oral health (self-assessed and determined by proffered correct responses concerning seven statements on oral health). Based on the score achieved by the participant, the level of oral health knowledge was categorized as very good/good, limited, and insufficient/none.

Foodstuffs enumerated in the category of sweets and sweet snacks of the FFQ questionnaire (as mentioned in the FFQ sweets and snacks category) were categorized—for the purposes of simplicity—in the following manner: (i) chocolate; (ii) cakes, ice cream, cookies, biscuits, doughnuts; (iii) sugar/sweets and candies; (iv) jams, syrups, and honey; (v) sugar-sweetened beverages SSBs and sweetened juices; (vi) sugar added to tea or coffee. With a view to performing the multivariate logistic regression analysis, the results were stratified into two groups—adolescents that rarely or never consume the sweets and the ones who do it often. Also, what was taken into consideration was the questions regarding healthy diets that consist of non-cariogenic products, such as milk, dairy, fruit and vegetables, cheese, yoghurt, milk, mineral water, and sugar free chewing gum.

The analysis of the socio-economic status (SES) included two variables: parents’ education and the economic status. Parents’ education was formed in five categories: primary (elementary), vocational, secondary, high or baccalaureate, no education. The respondents did not reveal their household’s monthly income, which is why the answers regarding the socio-economic status were divided on the basis of just one variable—a subjective assessment of a respondent—into the following: below/above average, average, could not say.

The scope of oral-health-related education in the dental office was concerned with the condition of the teeth and the gums, proper cleaning of teeth, recommended type of toothpaste and fluoridated products, dietary advice, and the frequency of check-up appointments.

The authors of the study devised a survey-administration procedure. The questionnaires were edited in Polish and were to be self-completed and administered at the beginning of a school year. The questionnaire was anonymous and distributed in paper form. It contained a short explanation of the aim of the study. At first, each participant was asked to sign informed consent and give an authorization to use the collected data. Full confidentiality was guaranteed to all the participants.

The data for the present study were collected from eighteen-year-olds at the time when the first semester of their school year just started and check-ups in dental offices started to avoid recall bias if the last dental appointment was not recent. Respondents then may not have remembered what they had learned at a dental visit.

The Strengthening the Reporting of Observational Studies in Epidemiology (STROBE) guidelines for reporting observational studies was used and followed [26].

### 2.4. Statistical Analysis

Survey results were subjected to the statistical analysis conducted using Statistica 10 software and the R 3.2 software package. In order to determine the relationships between pairs of variables, Spearman’s rank correlation analysis was performed. The use of the Spearman rank correlation coefficient, in addition to determining the significance of the relationship, also allowed for the interpretation of the strength of such a relationship and its type.

In order to assess the different factors on the oral health behaviors in adolescence, a bivariate logistic regression analysis was conducted, in which each individual factor was considered, as well as that of multivariate logistic regression, in which several factors were simultaneously assessed. Based on logistic regression, odds ratios (ORs) were defined for a relative chance of individual behaviors, including confidence intervals (with a confidence level of 95%). Adjusted odds ratios (AOR) were calculated with socio-economic factors as confounding factors, A1OR—at least one dental appointment in the past 12 months—and A2OR—at least two dental appointments in the past 12 months. The level of significance was assumed at *p* ≤ 0.05.

## 3. Results

### 3.1. Demographics of the Studied Group

**Figure 1:**
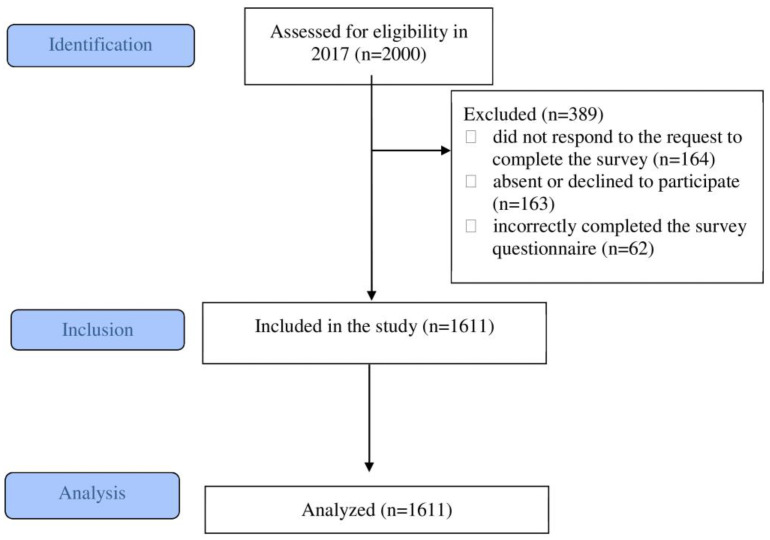
The study enrollment.

Out of 2000 adolescents originally invited to participate in the study, 1611 were included. The response rate was 81%. Figure 1 shows the study enrollment.

The examined sample of 1611 individuals consisted of 52.6% females, while 50.5% were rural dwellers. Table 1 focuses on the respondents’ characteristics: socio-economics, self-assessed knowledge of dental issues, and basic health behaviors. All of the eighteen-year-olds had the same educational level, since all participants attended high schools. The percentage distribution of participants from each school was very similar. The family’s economic status was usually assessed as “average” (Table 1).

### 3.2. Oral Health Knowledge and Health Behaviors

A very good perception of their gums and teeth was observed in 23.6% and 15.4% of respondents, respectively, with a good perception by 49.8% and 50.3%. No significant differences were noted in the self-assessment made by girls and boys. The knowledge of oral health issues was self-assessed as limited, with females’ understanding of this topic being considerably better than that of males’.

The lowest percentage of correctly recognized oral health statements concerned the benefits of fluoride application and the significance of a diet (Table 2). Frequent hygienic neglect and the consumption of foodstuffs with sugar, especially sweetened tea, were reported, with 14.7% of young people indulging in more than three snacks a day. Unfortunately, 37% of eighteen-year-olds admitted that they smoked cigarettes. Every fifth eighteen-year-old did so every day or several times a week.

The respondents claimed that they had obtained dental knowledge primarily from a dental team (897/55.7%) and family members (339/21.0%), followed by the internet, television and the radio (262/16.3%), and—rarely—teachers (54/3.4%) and others. Only education received in the dental office was statistically significantly correlated with the level of self-assessed dental knowledge of the respondents (r = 0.096; *p* < 0.05). The timing of the last dental visit was elicited. The number of respondents who failed to attend at least one dental visit in the past 12 months was 399 (24.7%), two—330/20.5%, three—194/12.0%, four and more—308/19.1%, with no significant differences between urban and rural regions (*p* = 0.432). Females visited a dentist more frequently than males, which was statistically significant (*p* < 0.001). Paid appointments in private dental offices were attended by 894 respondents (55.5%). A statistically significant relationship was found between private appointments and education provided by a dental team (r = 0.053; *p* < 0.05).

### 3.3. Health Education in the Dental Office and the Correlation with the Respondents’ Knowledge

The scope of oral-health-related education in the dental office most frequently concerned the condition of the teeth and the gums and the frequency of check-up appointments. Approximately half of the respondents were provided with such information (Table 3). Only one in ten respondents was given a recommendation regarding specific fluoridated products and obtained the instruction on healthy nutrition. Spearman rank correlation analysis revealed a significant, positive effect of education conducted during appointments on participants’ dental self-assessed knowledge, which the survey confirmed in both self-assessed statements and questionnaire verification (Table 3). Dietary advice was given to just 10.8% of the respondents. Providing such advice increased self-assessed oral health knowledge and the number of correct answers.

### 3.4. Correlations of the Frequency of Foodstuff Consumption and Hygienic Behaviors

Oral-health-related education provided by a dental team in the office and what the young people know about dental issues also positively influenced dietary and hygienic behaviors, which was statistically significant (Table 4 and Table 5). Parents’ education and economic status were also associated with the health behaviors of the youths. Socio-economic factors were positively correlated with favorable behaviors, but negatively with the consumption of cariogenic foodstuffs, except for jams and syrups/honey, where a positive correlation was observed for both mothers and fathers. The correlations were weaker for the economic status than for the education status and oral health knowledge.

Table 6 shows information conveyed by a dental team concerning oral health statistically influencing the occurrence of individual health behaviors related to socio-economic factors and gender (AOR), attending at least one visit in the dental office in the past 12 months (A1OR), and attending at least two dental visits in the past 12 months (A2OR). The dentist’s assessment of teeth and gum condition did not encourage the consumption of fresh fruit and vegetables, which would be more frequent than once a week. A significant relationship was observed when there were at least two dental visits in the past 12 months (A2OR) (Table 6).

## 4. Discussion

In this cross-sectional survey study conducted among adolescents at the age of eighteen, data regarding nutritional and oral-health-related knowledge, as well as self-reported behavior, were collected in order to compare the awareness with everyday practices, without any intervention.

To the best of our knowledge, the present study is the first to investigate the oral-health-related knowledge and behaviors of eighteen-year-olds in all provinces in Poland. In addition, not too much research has been performed so far on the oral health behaviors of adolescents as a complex phenomenon. Epidemiological data regarding the oral health of eighteen-year-olds in Poland indicate that there is a clear need to educate them about oral health [18]. The present research confirms insufficient knowledge and the high rate of unhealthy behaviors related to the oral cavity among eighteen-year-olds. Generally, knowledge and daily practices were not congruent in our study. Admittedly, the only measure of the examined behaviors was the answers to the survey questions, which may lower the value of the results. It transpired, however, that the percentage of respondents replying to similar questions in the past in one province of Poland was similar [17]. Also, 22.2% of young American adults exhibited “low” knowledge [10].

Nearly all respondents positively evaluated the condition of their dentition, with 93.3% claiming that it was very good, good, or average, even though the frequency of caries in eighteen-year-olds in Poland was 93.2%, 6.50 teeth were affected with the disease, and two teeth on average required treatment [18]. Research conducted on adolescents in Brazil, Portugal, Romania, and Sweden also demonstrated an over-optimistic self-assessment of the oral health in comparison with the results of the clinical examination [27,28].

Many adolescents’ habits did not specifically meet the American Academy of Pediatric Dentistry (AAPD) guidelines of oral health, including diet, oral hygiene management, and professional preventive care [1]. Many teenagers often eat snacks and excessive amounts of refined carbohydrates and beverages, which contain acids and sugars in the form of fizzy drinks, energy drinks, junk food, and coffee [1,4,29,30,31,32]. The risk of caries increases significantly with more than three sugary snacks/day [1]. High intake of sugars has been identified not only with higher energy intakes, but also with diets of lower nutritional quality junk food.

In the sample examined in this study, dietary advice was given to just 10.8% of the respondents. There was a relationship between self-assessed knowledge of oral health issues and dietary behaviors. Studies suggest a link between frequent snacking and some diseases, especially being overweight/obese and dental caries [31,33]. The frequency of snacking, its share in daily energy intake, and snacks’ influence on the energy requirement increased considerably, together with the shift from main meals to snacks [31]. Only 40% of our respondents understood that frequent snacking between meals promotes the development of dental caries. The study on the snack consumption of Canadians found that 54% of adolescents consumed a snack 2–3 times a day, with 11% eating snacks four or more times a day [31]. The results are in line with our findings. Only 14.7% of our respondents eat snacks >3 times a day, with no differences between gender groups. In our study, the most frequently consumed healthy snacks were cheese, yoghurt, and milk (63%). The percentage was significantly higher among females. Our results are slightly different from other studies [29,32]. Only 14.9% of the Chinese and 19.6% of Spaniards consumed milk or yoghurt [29,32]. Dairy products (DPs) are regarded as an indispensable element of teenagers’ diets since they have a positive influence on the health of the oral cavity, promoting enamel remineralization and reducing demineralization, stimulating salivary flow, and raising salivary calcium and phosphate levels and the buffering capacity of saliva. In addition to that, DPs positively affect the oral microbiota and the immune response. Therefore, they protect the enamel and have an impact on the susceptibility to caries. On the other hand, certain sweetened DPs may contain added sugar, which is cariogenic. This is why it is necessary to differentiate between the cariogenic potential of sugars in those products and the non-cariogenic ingredients of milk [33].

The foods that followed dairy products in our study were vegetables and fruit (49.6%), another major source of intrinsic sugars, which was also slightly different from other studies. The fruit consumption in Spanish adolescents was 20% [32]. Also, Canadians were more likely to select vegetables and fruit, as well as milk and dairy products, as snacks [31]. Only 8.8% of Brazilian adolescents followed the guidelines regarding the daily intake of fruit and vegetables [4]. Silva et al. [34] revealed that 88.6% of teenagers consumed insufficient amounts of fruit and vegetables. The consumption of fresh fruit is connected with a shorter time of exposure in the oral cavity and the presence of fructose, which is less cariogenic than sucrose. The presence of fiber and polyphenols in fruit was associated with the disruption of plaque formation and lowering the acidity of oral bacteria. Food that is rich in nonmilk extrinsic sugars (NMESs) should be replaced with fresh fruit and vegetables [35]. Individuals who consume relatively higher amounts of fresh fruit are not affected by caries [35]. Clinical studies have confirmed the effectiveness of fruit in caries prevention; however, the results are inconsistent and inconclusive. Some authors suggest that the sensible consumption of fruit between meals does not promote cavities [35].

As the preference for sweets is higher among teenagers than adults, adolescence is a critical period during which individuals are more susceptible to diseases associated with a high intake of sugar, including obesity, coronary heart disease (CHD), diabetes, and caries. Adolescents consume more sugar than recommended, which may be explained by the lack of knowledge about food containing sugar, as well as the influence of a variety of factors, such as the living environment and the availability of sweetened food products [29]. The European Food Safety Administration (EFSA) found that in adolescents, mean intakes of total sugars ranged from 15 E% in Italians to 27 E% in Estonians and Finns [36]. Mean intakes of added sugars ranged from 5 E% in Cypriot males to 16 E% in Dutch males. The major contributors to mean added sugar intake were sugars and confectionery (from 13% in Portugal to 56% in Finland) and SSBs (from 7% in Latvia to 41% in the Netherlands) [36]. The findings of the present study showed that adolescents’ daily use of sweetened tea was higher (42.6%) than that of other beverages. Apart from coffee, their main sources of added sugar included sweetened coffee (19.7%), SSBs (21.5%), and sweetened juices (20%) at least once or a few times a day, which significantly increases the level of fermentable carbohydrates in the oral cavity. Sweetened coffee (pH of 4.9–5.2) consumption, due to the fact that it lowers the pH level of saliva, can cause a variety of dental health issues, including caries, tooth erosion, and periodontal disease. In comparison with nationwide studies conducted in Poland in 2014, drinking coffee with sugar every day or a few times daily decreased only slightly (from 24.2% to 19.7%), the consumption of SSBs fell from 27.1% to 21.5%, and the intake of sweets and candies remained at the same level (23.7%) [18]. Our results are in line with the previous studies [29].

We also found that males tend to drink more SSBs, sweetened juices, and energizing beverages. Findings similar to ours were observed in other pieces of research [29,30,31,32,37,38]; 34.2% of Canadian participants still self-reported drinking SSBs every day [31]. In U.S. studies, about half of the population of the young adults aged 20 would drink sugar-sweetened beverages (SSBs) at least once a day, with 7.5% indulging in this practice three or more times a day, which constituted more than 6% of the daily energy demand [37]. The high frequency of the consumption of SSBs increases plaque acidity and the potential for plaque formation and bacterial growth in the oral cavity [36]. The consumption of SSBs by young adults is associated with a risk of caries, which is—in turn—the main cause of tooth loss [38]. Kim et al. [38] observed that 1 in 4 young adults in the United States reported having lost at least one permanent tooth due to dental caries, and almost 2 out of 5 young adults reported drinking SSBs at least once a day. As evidenced by the monitoring data of the Polish population, every tenth individual at the age of 18 has at least one tooth missing due to caries or its complications [18].

The second main source of added sugars in our study was sugar/sweets and candies (23.7%), which is similar to the results of other investigations [29,30,32]. The study of Austregésilo et al. [4] revealed a high intake (once or more daily) of sweets (82.8%), SSBs (75.2%), and carbohydrate-rich food (75.5%) among Brazilian adolescents. National Health and Nutrition Examination Survey (NHANES) data indicated that snacks and sweets constituted 31% of the total intake of added sugars [39]. The WHO recommends reducing the consumption of sugars to less than 10% of total energy intake and suggests a further reduction of the ingestion of free sugars to below 5% of total energy intake [40]. Generally speaking, public health programs that aim at the reduction of sugar intake must concentrate on this vulnerable group. It is necessary to improve the food environment in schools by means of the regulation of the sales of food products and beverages that are high in simple sugars.

Researchers who study gender-related health behaviors stress that young females tend to manifest oral-health-related attitudes more frequently [40,41,42,43]. It is believed that girls are usually better informed, and therefore, they pay more attention to behaviors related to oral health [40,41,42,43]. This is corroborated by our findings. Regardless of the respondents’ gender, the whole sample demonstrated major knowledge gaps and misinterpretations, reflecting reports by other authors [44]. In the present study, every second respondent claimed to have knowledge about the preventative role of fluoride. In the 1998–2014 Polish nationwide survey, the percentage of eighteen-year-old respondents who knew about the benefits of fluoride prophylaxis ranged from 29% in 1998 to 54.2% in 2014 [16]. Similarly to Poland, adolescents from North West of England had unsatisfactory self-assessed knowledge of the role of fluoride [44].

As is commonly known, the absence of knowledge and skills constitutes a certain barrier in a positive display of oral-health-related behaviors. An important factor that modifies them is the sense of one’s self-agency in caring for oral health. In the studied sample, there was a relationship between self-assessed knowledge and hygienic behaviors. Only 67.8% of adolescents described habits that specifically met the AAPD [1] guidelines about brushing twice daily. In 2014, brushing teeth at least once a day was reported by 63.4% of the respondents [17]. Similarly, in Norway, 66.8% of eighteen-year-olds brushed their teeth twice a day and 14.2% used dental floss at least once a day [41]. Slightly different results were obtained by Ericsson et al. [42]; 76% of 506 nineteen-year-old Swedes brushed their teeth at least twice a day, and only 4% used dental floss daily. In the study of North-East Italian adolescents (mean age 17 years old) these percentages were 83% and 7%, respectively [43]. It has to be stressed that as many as 61.3% of the study subjects had never used dental floss. Nasir and Vu [41] observed that persons declaring that they brush teeth twice a day and also floss understood the benefits of these activities. In the present study, the importance of self-assessed dental and gingival health was not analyzed. What was assessed, however, was the character of information relayed to the respondents by a dental team. Practical knowledge related to oral hygiene was positively correlated with correct hygienic behaviors, just like the advice on a cariostatic diet with the consumption of a sugar-free diet. The highest correlation coefficients were revealed between hygienic and dietary behaviors and information on the condition of the teeth and gums conveyed by the dental practitioner.

Investigations of a variety of health behaviors, including oral hygiene practices, based on a health belief model (HBM), indicate that what we know about the susceptibility to oral diseases and their consequences, as well as the belief in the ability to fulfill the recommended preventative measures, promotes compliance with these recommendations [8,41].

In Polish dental practices, dentists (or sometimes hygienists) usually provide oral health information to patients. Høiseth and Jasbi [3] highlight the key role that a dental team can play in oral health promotion. The participants of their study appreciated dental professionals who take the time to motivate and make recommendations. According to the assumptions made by FDI World Dental Federation’s (FDI) Vision 2030 Working Group, healthcare employees will possess knowledge and skills that will enable them to successfully prevent and treat oral diseases [45].

Nutrition education is one of several components of education strategies for health improvement. Regarding dietary behaviors, the authors of the present study noticed more correlations between the consumption of foodstuffs/self-assessed knowledge/test-verified knowledge when compared with dietary education. In the logistic regression model, conveying dietary recommendations increased the chance of using sugar-free chewing gum; however, it was only after socio-economic factors were introduced to the statistical model as confounding factors. Many authors have reported the significance of factors, such as gender, parents’ education, or economic status, which is in line with the Spearman analysis performed for the purpose of this study [1,42,46,47,48]. Only every tenth respondent received dietary recommendations during a dental visit. This low number can be attributed to a few factors. One of the reasons is the fact that adolescents might feel discomfort or embarrassment while discussing their dietary habits. They may also believe that such a consultation concentrates mostly on the condition of their teeth and treatment, which results in them placing priority on the discussion about their dentition and routine oral care and not their dietary choices. Høiseth and Jasbi [3] provide another explanation, claiming that teenagers may not entirely understand the impact of their diet on their oral health. As a result, they perceive it as less significant and emphasize more evident aspects related to oral health.

Cigarette smoking still constitutes a common risky behavior among adolescents and may negatively influence oral health. This problem among Polish teenagers was discussed in a previous paper [49].

As far as teenagers are concerned, education needs to be repeated. According to the literature, as least four contacts with the target group are required to increase the chance of success [50]. In Poland, an oral examination once a year is mandatory during the high school period. Regular dental check-ups, at least twice a year, are the individual’s responsibility. Thus, it is very important to encourage eighteen-year-olds to undergo regular dental check-ups. In the present study, the introduction of “the frequency of dental visits” factor into the statistical model revealed an increased chance of proper hygienic behaviors and the use of sugar-free chewing gum. Also, what emerged was a statistical significance related to the consumption of fruit and vegetables at least once a day.

Regular users of health care services have a better chance of knowledge acquisition/skills improvement and to be motivated towards the modification of their oral-health-related behaviors, hence reducing the probability of the occurrence of health issues [51]. In the present study, it was the dental team that was the main source of information related to oral health. Therefore, the dramatically low percentage of teenagers who received dietary and hygienic advice and recommendations to prevent dental caries should be regarded as worrying. Admittedly, the authors relied on questionnaire data, which can be burdened with a certain risk of untrue answers, but these results concur with those of other authors’ and with earlier epidemiological results in Poland. Studies of Italian teenagers revealed that 60.1% of this population received information on oral hygiene exclusively from parents, 8.5% from the dental team and 23.0% from both [43]. In the British studies, it is the families that prevail in influencing personal attitudes and oral health views of adolescents [44]. At the same time, these authors blamed the parents for insufficient involvement in preventing caries. A dental practitioner was perceived as the one who endorses oral health related oral behaviors promoted by the parents. Young people, however, voiced their concerns regarding the quality of the education presented by the dentist and dental team, which the observations in the present study seem to confirm. They complained that they would be given the same piece of advice on every visit without accounting for individual needs or the verification of results. The analysis of correlations performed in the present study indicated a significant relationship among education given in the dental office. Low values of correlation coefficients reveal the insufficient effectiveness of these activities. An intervention that is noted to be more effective than the traditional oral health education (OHE) for adolescents was motivational interviewing, which was a person-centered counseling strategy [14]. Studies conducted on a sample of dental teams in Norway revealed that only 61.2% applied motivational interviewing (MI) in their adolescent patients [52]. At the same time, only 40% stated that they were familiar with national guidelines regarding the use of MI with adolescents, and only 25% applied it regularly. On top of that, more than half of the respondents believed that some teenagers would never change their attitudes, regardless of the efforts of the dental team [52].

The training needs of dentists and their staff should be discussed. In most countries, universities/dental faculties organize continuing dental education and activities in collaboration with National Dental Associations (NDAs) [53]. Continuing Professional Development (CPD) is mandatory in most European countries [54]. In Poland, the postgraduate and specialist education of doctors does not solve problems, such as the availability of courses and residency programs [55]. More educational and training offers should be provided to dentists so that good standards and a high quality of dental care could be ensured.

This study has a number of strengths, such as a large number of participants, high response rate, and detailed qualitative data, which made it possible to carefully evaluate the respondents’ answers. The information obtained can be useful in information campaigns and could help shape the dentists’ attitudes to educating the youths on oral health behaviors. The survey was conducted in government-run schools, and the population studied was relatively homogeneous and represents a large social structure without the inclusion of different cultures and ethnicities. This is, therefore, a nationally-representative survey. A population sample was used, contrary to a clinical convenience sample. The presence of controlling confounding factors, such as socio-economic ones, was taken into consideration and analyzed so that their influence on the results could be determined. The educational environment and participants’ experiences might have differed depending on the school, which may have impacted the results of the research presented in this article. On the other hand, only high school students were involved in the study. Nevertheless, the present study collected the data from eighteen-year-olds at the time when the first semester of their school year just started. With a female-to-male ratio and urban-to-rural ratio of 1:1 in the respondents, there was minimal risk of sampling bias in the present study. Furthermore, this study was conducted following STROBE guidelines for reporting observational studies.

### Limitations

The presented results should be interpreted by taking into account certain limitations. Data were gathered though self-reporting, and the participants might not have provided honest answers. In addition, their responses might have been biased due to the excessive reporting of certain habits or behaviors that seem to send warnings about their health. Data provided by the analyzed group might have been distorted, which may have led to the information bias that consists in the overestimation of health-promotion behaviors and the underestimation of those that are risky. What is more, the survey was conducted during one appointment, while it would be necessary to carry out a long-term observation in order to determine the consistency of the responses regarding their oral health. Certain questions could also have confused the participants as far as their knowledge and behavior are concerned. If the last dental appointment did not take place recently, there might have occurred a bias related to recalling what they had learned from a dental team during that visit. Another limitation of the study is the fact that it was impossible to investigate the influence of missing data on reported results. Due to ethical reasons, it was settled that participants with missing data did not give their consent to data use. This is why the data collected were entirely excluded from the analysis. It is acknowledged that the estimations of individuals having only full data at their disposal might be biased. Finally, since it was a cross-sectional study, it made it possible to determine only the relationship and not cause-and-effect associations between variables.

The described study was conducted in 2017, as major changes have occurred over the past 7 years (for example, the COVID19 epidemic), which may have altered the characteristics identified in the study.

As alcohol and drug use represent a major concern of public health among adolescents, the authors will consider, in a future study, examining the impact of alcohol and drug use in adolescents on increasing the susceptibility to oral and dental disease. These aspects were not taken into account in the study. The use of alcohol and illicit drugs are risk factors for poor oral health outcomes in adolescents that can extend into adulthood and lead to substance misuse or abuse and behaviors in adulthood that may have negative oral health outcomes as well [56]. For example, heavier alcohol use is also associated with high-risk oral HPV among older adolescents and young adults [57,58].

## 5. Conclusions

The results have highly important implications from the perspective of public health. The evaluation of the oral-health-related knowledge and habits of adolescents is necessary to fully understand the needs of the society regarding oral health. Nearly all participants positively assess the condition of their teeth, even though the prevalence of caries is 93.2%. Around two-thirds of adolescents brush their teeth twice daily and use dental services.

Most adolescents regard dental health as important and claim that they possess adequate knowledge, although their practices may not follow AAPD guidelines. This study demonstrated that oral health knowledge and the associated behaviors of adolescents, as well as the involvement of the dental team in education and prophylactics, are inadequate. The scope of oral-health-related education in the dental office most frequently concerned the condition of the teeth and the gums and the frequency of check-up appointments. A recommendation regarding the instruction on healthy nutrition/dietary advice and specific fluoridated products was insufficient. Education and instruction in the dental office have a favorable influence on oral-health-related behaviors. Providing such recommendation increased self-assessed oral health knowledge. It is, therefore, necessary to undertake systemic solutions so that dental practitioners are more involved. Providing oral health knowledge should be the standard of care.

The present study reveals the challenges and detailed information regarding the improvement of adolescents’ health in Poland. In order to effectively enhance the knowledge, approach, and practices related to oral health, there is a need for oral health education (OHE) programs and interventions.

## Figures and Tables

**Table 1:** Socio-economic characteristics of the respondents.

Parameters		n (%) 1611 (100%)
Respondents’ gender	female	847 (52.6)
	male	764 (47.4)
Region of residence	urban	797 (49.5)
	rural	814 (50.5)
Mother’s education status	elementary	47 (2.9)
	vocational	418 (25.9)
	secondary	532 (33.0)
	higher or baccalaureate	446 (27.7)
	no education	168 (10.4)
Father’s education status	elementary	55 (3.4)
	vocational	566 (35.1)
	secondary	457 (28.4)
	higher or baccalaureate	317 (19.7)
	no education	216 (13.4)
Family’s economic status	below average	47 (2.9)
	average	893 (55.4)
	above average	382 (23.7)
	could not say	289 (17.9)

**Table 2:** Oral health knowledge and health behaviors of eighteen-year-olds related to their gender.

Parameters		Total	Gender		
			Female	Male	*p*
		n (%) 1611 (100%)	847 (52.6%)	764 (47.4%)	
Self-assessed oral health knowledge	very good/good	563 (34.9)	333 (39.3)	230 (30.1)	<0.001 *
	limited	1016 (63.1)	502 (59.3)	514 (67.3)	<0.001 *
	insufficient/none	32 (2.0)	12 (1.4)	20 (2.6)	0.085
Correct responses	1. In children and adolescents dental caries develops faster than in adults.	1032 (64.1)	603 (71.2)	429 (56.2)	<0.001 *
	2. If parents have many carious lesions, then their children will also have many cavities.	1124 (69.8)	615 (72.6)	509 (66.6)	0.009 *
	3. Fluoride compounds penetrate the enamel, making it resistant to caries.	787 (48.9)	430 (50.8)	357 (46.7)	0.105
	4. There are dental surfaces that cannot be cleaned with a toothbrush, and flossing is required.	1310 (81.3)	741 (87.5)	569 (74.5)	<0.001 *
	5. Frequent sugar snacks between meals favor the development of dental caries.	656 (40.7)	348 (41.1)	308 (40.3)	0.753
	6. Fruit juices and sugar-sweetened beverages may damage teeth.	1236 (76.7)	685 (80.9)	551 (72.1)	<0.001 *
	7. Adolescents should do dental check-ups at least once a year.	1397 (86.7)	771 (91.0)	626 (81.9)	<0.001 *
Brushing teeth at least twice daily		1092 (67.8)	690 (81.5)	402 (52.6)	<0.001 *
Using dental floss		624 (38.7)	415 (49.0)	209 (27.4)	<0.001 *
Intentional use of fluoridated toothpaste		639 (39.7)	351 (41.4)	288 (37.7)	0.125
Using fluoride mouth rinses		819 (50.8)	486 (57.4)	333 (43.6)	<0.001 *
Snacking > 3 times a day		237 (14.7)	91 (10.7)	146 (19.1)	<0.001 *
Foodstuffs consumed daily or several times a day	chocolate	58 (3.6)	23 (2.7)	35 (4.6)	0.041 *
	cakes, ice-cream, biscuits sponge, pastries, doughnuts, layer cakes	216 (13.4)	117 (13.8)	99 (12.9)	0.102
	sugar/sweets and candies	382 (23.7)	204 (24.1)	178 (23.3)	0.108
	jams and syrups/honey	100 (6.2)	43 (5.1)	57 (7.5)	0.066
	sugar-sweetened beverages (SSBs)	346 (21.5)	142 (16.8)	204 (26.7)	0.070
	sweetened juices	322 (20.0)	156 (18.4)	166 (21.7)	0.924
	tea with sugar	686 (42.6)	346 (40.9)	340 (44.5)	0.787
	coffee with sugar	320 (19.7)	163 (19.2)	157 (20.6)	0.692
	energy drinks	112 (6.9)	43 (5.01)	69 (9.0)	0.008 *
	potato chips	141 (8.7)	55 (6.5)	86 (11.3)	0.003 *
	chewing gum with sugar	350 (21.7)	197 (23.2)	153 (20.0)	0.001 *
	fresh fruit and vegetables	799 (49.6)	461 (54.4)	338 (44.2)	<0.001 *
	cheese, yoghurt, milk	1015 (63.0)	560 (66.1)	455 (59.6)	0.001 *
	mineral water	1297 (80.5)	716 (84.5)	581 (76.1)	<0.001 *
	chewing gum without sugar	328 (20.4)	201 (23.7)	127 (16.6)	<0.001 *

* Statistical significance, *p* ≤ 0.05.

**Table 3:** Health education by a dental team during visits in the dental office and the correlation with the respondents’ knowledge.

The Scope of Dental Team Education	Respondents Educated by Dental Team n/% 1611 (100%)	Spearman’s Rank Correlation Coefficients (r)						
		Self-Assessed Oral Health Knowledge	The Number of Correct Responses Concerning Oral Health	The Number of Correct Responses Concerning Oral Health (by Individual Statements)				
				Question 2 n (%) 1124 (69.8%)	Question 3 n (%) 787 (48.9%)	Question 4 n (%) 1310 (81.3%)	Question 6 n (%) 1236 (76.7%)	Question 7 n (%) 1397 (86.7%)
Dentist’s assessment of teeth and gums condition	931 (57.8)	0.154 * p < 0.001	0.129 * p < 0.001	0.083 * p = 0.013	0.028 *p* = 0.850	0.100 * p < 0.001	0.071 * p = 0.019	0.117 * p < 0.001
Frequency of dental check-ups	756 (46.9)	0.120 * p < 0.001	0.194 * p < 0.001	0.110 * p < 0.001	0.101 * p < 0.001	0.128 * p < 0.001	0.091 * p = 0.008	0.159 * p < 0.001
Proper cleaning of teeth	499 (31.0)	0.065 * p = 0.019	0.076 * p = 0.013	0.017 *p* = 0.900	0.054 * p = 0.030	0.073 * p = 0.018	0.035 *p* = 0.769	0.064 * p = 0.019
Recommended type of toothpaste	326 (20.2)	0.100 * p < 0.001	0.087 * p = 0.004	0.019 *p* = 0.874	0.061 * p = 0.021	0.059 * p = 0.022	0.058 * p = 0.022	0.092 * p = 0.001
Recommended fluoridated products	144 (8.9)	0.085 * p = 0.004	0.043 *p* = 0.087	−0.016 *p* = 0.899	0.051 * p = 0.023	0.011 *p* = 0.929	0.023 *p* = 0.833	0.001 *p* = 0.999
Dietary advice	146 (9.1)	0.075 * p = 0.013	0.075 * p = 0.013	0.015 *p* = 0.985	0.051 * p = 0.023	0.035 *p* = 0.102	0.046 *p* = 0.098	0.015 *p* = 0.985

* Statistical significance, *p* ≤ 0.05.

**Table 4:** Correlations of the frequency of foodstuff consumption with socio-economic factors, dental knowledge, and oral-health-related diet education provided by a dental team during dental appointments.

Frequency of Foodstuff Consumption:	Education Status		Oral Health Knowledge		The Scope of Dental Team Education		Economic Status
	Mother’s	Father’s	Self-Assessment	The Number of Correct Responses in the Test of Oral Health	Dental Assessment of Condition of Teeth and Gums	Instruction on Cariostatic Diet	
Chocolate	−0.067 *	−0.078 *	−0.057 *	−0.123 *	−0.007	−0.037	−0.028
	*p* = 0.028	*p* = 0.018	*p* = 0.047	*p* < 0.001	*p* = 0.902	*p* = 0.784	*p* = 0.453
Cakes, ice-cream, biscuits, sponge, pastries, doughnuts, layer cakes	−0.112 *	−0.107 *	−0.123 *	−0.087 *	−0.079 *	−0.067 *	−0.054 *
	*p* < 0.001	*p* < 0.001	*p* < 0.001	*p* = 0.009	*p* = 0.021	*p* = 0.030	*p* = 0.046
Sugar/sweets and candies	−0.067 *	−0.056 *	−0.058 *	−0.067 *	0.026	0.015	−0.066 *
	*p* = 0.027	*p* = 0.046	*p* = 0.043	*p* = 0.026	*p* = 0.754	*p* = 0.893	*p* = 0.025
Jams and syrups/honey	0.074 *	0.071 *	0.063 *	0.057 *	0.018	0.029	0.058 *
	*p* = 0.001	*p* = 0.010	*p* = 0.033	*p* = 0.046	*p* = 0.894	*p* = 0.778	*p* = 0.047
Sugar-sweetened beverages (SSBs)	−0.062 *	−0.068 *	−0.100 *	−0.109 *	−0.049	−0.003	0.013
	*p* = 0.033	*p* = 0.029	*p* < 0.001	*p* < 0.001	*p* = 0.068	*p* = 0.999	*p* = 0.901
Tea with sugar	−0.103 *	−0.077 *	−0.017	−0.082 *	0.004	0.024	−0.073 *
	*p* < 0.001	*p* = 0.010	*p* = 0.630	*p* = 0.006	*p* = 0.999	*p* = 0.371	*p* = 0.011
Coffee with sugar	−0.041	−0.016	−0.001	−0.087 *	−0.065 *	0.017	−0.021
	*p* = 0.098	*p* = 0.888	*p* = 0.999	*p* = 0.005	*p* = 0.031	*p* = 0.900	*p* = 0.389
Energy drinks	−0.005	0.046	−0.038	−0.159 *	−0.053 *	0.023	0.032
	*p* = 0.996	*p* = 0.087	*p* = 0.282	*p* < 0.001	*p* = 0.048	*p* = 0.372	*p* = 0.703
Fresh fruit and vegetables	0.087 *	0.131 *	0.126 *	0.081 *	0.100 *	0.054 *	0.090 *
	*p* = 0.007	*p* < 0.001	*p* < 0.001	*p* = 0.008	*p* < 0.001	*p* = 0.041	*p* = 0.001
Cheese, natural yoghurt, milk	0.05	0.049	0.068 *	0.092 *	0.072 *	0.086 *	0.056 *
	*p* = 0.061	*p* = 0.058	*p* = 0.029	*p* = 0.002	*p* = 0.014	*p* = 0.035	*p* = 0.041
Mineral water	0.082 *	0.117 *	0.110 *	0.166 *	0.097 *	0.057 *	0.060 *
	*p* = 0.009	*p* < 0.001	*p* < 0.001	*p* < 0.001	*p* = 0.001	*p* = 0.046	*p* = 0.040
Sugar-free chewing gum	0.056 *	0.079 *	0.133 *	0.032	−0.006	0.071 *	0.062 *
	*p* = 0.041	*p* = 0.010	*p* < 0.001	*p* = 0.703	*p* = 0.993	*p* = 0.010	*p* = 0.033

* Statistical significance, *p* ≤ 0.05.

**Table 5:** Correlations of hygienic behaviors of adolescents in view of socio-economic factors, self-assessed dental knowledge, and oral health education provided by a dental team during dental appointments.

Hygienic Behaviors	Education Status		Economic Status	Oral Health Knowledge		The Scope of Dental Team Education				
	Mother’s	Father’s		Self-Assessment	Number of Correct Responses	Dentist’s Assessment of Condition of Teeth and Gums	Proper Cleaning of Teeth	Flossing	Recommended Type of Toothpaste	Recommended Specific Fluoridated Products
Brushing teeth ≥ 2 times a day	0.064 * p = 0.048	0.069 * p = 0.045	0.041 *p* = 0.098	0.126 * p < 0.001	0.149 * p < 0.001	0.113 * p < 0.001	0.042 *p* = 0.193	0.066 * p = 0.037	0.027 *p* = 0.773	0.016 *p* = 0.903
Intentional use of fluoridated toothpaste	0.044 *p* = 0.402	0.032 *p* = 0.650	0.070 * p = 0.019	0.138 * p < 0.001	0.261 * p < 0.001	0.107 * p < 0.001	0.102 * p < 0.001	0.065 * *p* = 0.029	0.100 * p < 0.001	0.160 * p < 0.001
Using fluoride mouth rinses	0.054 * *p* = 0.047	0.093 * p = 0.001	0.110 * p < 0.001	0.105 * p < 0.0001	0.102 * p < 0.001	0.082 * p = 0.003	0.057 * p = 0.401	0.106 * p < 0.001	0.140 * p < 0.001	0.099 * p < 0.001
Flossing	0.078 * p = 0.006	0.098 * p < 0.001	0.078 * p = 0.006	0.179 * p < 0.001	0.178 * p < 0.001	0.109 * p < 0.001	0.118 * *p* < 0.001	0.188 * *p* < 0.001	0.04 *p* = 0.999	0.028 *p* = 0.455

* Statistical significance, *p* ≤ 0.05.

**Table 6:** Information conveyed by dental team concerning oral health statistically influencing the occurrence of individual health behaviors related to socio-economic factors and gender (AOR), attending at least one visit in the dental office in the past 12 months (A1OR), and attending at least two dental visits in the past 12 months (A2OR).

Conveyed Information		Health Behaviors n (%)	OR	AOR	A1OR	A2OR
		Fresh fruit and vegetables ≥ once a day				
Dentist’s assessment of teeth and gums condition	Yes	923 (57.3)	1.01 (0.94–1.12) *p* = 0.547	0.99 (0.82–1.07) *p* = 0.567	1.13 (0.98–1.21) *p* = 0.637	1.21 (1.07–2.11) *p* = 0.003 *
	No	664 (41.2)				
		Sugar-free chewing gum				
	Yes No	108 (6.7) 942 (58.5)	1.15 (0.85–1.33) *p* = 0.122	1.22 (1.02–1.34) *p* = 0.034 *	1.56 (1.21–1.87) *p* = 0.010	1.78 (1.54–1.91) *p* = 0.003 *
		Tooth brushing ≥2 times a day				
Dentist’s assessment of teeth and gums condition	Yes	673 (41.8)	1.21 (1.10–1.46) *p* = 0.003 *	1.23 (1.15–1.38) *p* < 0.001 *	1.23 (1.17–1.33) *p* < 0.001 *	1.25 (1.17–1.31) *p* < 0.001 *
	No	419 (26.0)				
		Intentional use of fluoridated toothpaste				
Dentist’s assessment of teeth and gums condition	Yes	411 (25.5)	1.26 (1.05–1.55) *p* = 0.0193 *	1.35 (1.11–1.46) *p* = 0.004 *	1.45 (1.22–1.63) *p* < 0.001 *	1.49 (1.24–1.59) *p* < 0.001 *
	No	228 (14.2)				
Recommending specific fluoridated products	Yes	93 (5.8)	1.73 (1.24–2.21) *p* < 0.001 *	2.01 (1.89–2.67) *p* < 0.001 *	2.45 (2.12–2.67) *p* < 0.001 *	3.02 (2.67–3.21) *p* < 0.001 *
	No	546 (33.9)				
		Flossing				
Proper cleaning of teeth Flossing	Yes	236 (14.6)	1.36 (1.15–1.67) *p* = 0.001 *	1.45 (1.26–1.63) *p* < 0.001 *	2.11 (1.87–2.37) *p* < 0.001 *	2.09 (1.91–2.32) *p* < 0.001 *
	No Yes No	388 (24.1) 146 (9.1) 478 (29.7)				
			1.74 (1.30–2.12) *p* < 0.001 *	1.99 (1.47–2.42) *p* < 0.001 *	2.02 (1.89–2.33) *p* < 0.001 *	2.23 (2.01–2.39) *p* < 0.001 *

OR—odds ratios; AOR—an adjusted odds ratios with socio-economic factors as confounding factors, A1OR—at least one dental appointment in the past 12 months A2OR—at least two dental appointments in the past 12 months; * statistical significance, *p* ≤ 0.05.

## Data Availability

The original contributions presented in this study are included in the article. Further inquiries can be directed to the corresponding author on reasonable request following the approval of the Ministry of Health.

## Abbreviations

The following abbreviations are used in this manuscript:

DMFT	Decayed, Missed, Filled Teeth
FFQ	Food Frequency Questionnaire
WHO	World Health Organization
CMOHK	Comprehensive Measure of Oral Health Knowledge
HBM	The Health Belief Model
SES	Socio-Economic Status
STROBE	The Strengthening the Reporting of Observational Studies in Epidemiology
OR	Odds Ratio
AOR	Adjusted Odds Ratio
SSBs	Sugar-Sweetened Beverages
NMES	Nonmilk Extrinsic Sugars
CHD	Coronary Heart Disease
EFSA	The European Food Safety Administration
NHANES	National Health and Nutrition Examination Survey
AAPD	American Academy of Pediatric Dentistry
NDAs	National Dental Associations
CPD	Continuing Professional Development
